# Smoking, general and oral health related quality of life – a comparative study from Nepal

**DOI:** 10.1186/s12955-020-01512-y

**Published:** 2020-07-31

**Authors:** Reshu Agrawal Sagtani, Sunaina Thapa, Alok Sagtani

**Affiliations:** 1 School of Public Health, Patan Academy of Health Sciences, Lalitpur, Nepal; 2 Annapurna Neurological Institute and Allied Health Sciences, Kathmandu, Nepal; 3 Department of Oral and Maxillofacial Surgery, Kathmandu Medical College Teaching Hospital, Sinamangal, Kathmandu, Nepal

**Keywords:** Smoking, Perceived oral health, Quality of life, Nepal

## Abstract

**Background:**

Perceived dental health has shown to have a significant predictive effect on overall health perception and life satisfaction. Thus, it seems plausible that Health Related Quality of Life (HRQOL) measures are associated with Oral Health Related Quality of Life (OHRQOL) dimensions in Nepalese context as well. The adverse effects of tobacco on oral health are reported worldwide including Nepal. However, evidence which can quantify effects of tobacco smoking on dental health perception is limited. Thus, a study was designed to find association of smoking and socio demographic characteristics with OHRQOl and to determine association between OHRQOL and HRQOL among dental patients in Nepal.

**Methods:**

A cross sectional study was conducted among 125 current smokers and 125 non-smokers who attended oral surgery OPD of a teaching hospital in Kathmandu, Nepal. The study participants were enrolled through consecutive sampling and data was collected through a semi-structured questionnaire. The questionnaire consisted of questions related to sociodemographic characteristics, tobacco history, Oral Health Impacts Profile (OHIP)-14 and World Health Organization Quality of Life Brief version (WHOQOL-Bref) to assess OHRQOl and HRQOL respectively. Descriptive and inferential statistics were calculated by using SPSS version 18.0. The level of significance was set at 5%.

**Results:**

Among the socio demographic characteristics, patients with education of more than Class 12 had significantly higher average OHRQOL scores (*p* = 0.013) compared to illiterate patients. Current smokers reported significantly poorer scores in sub scales of psychological disability (*p* = 0.001), social disability (*p* = 0.003), physical pain (*p* < 0.001), functional limitation (*p* = 0.007) and also overall perceived oral health compared to nonsmokers. OHRQOL was significantly correlated with overall HRQOL in physical (*p* = 0.015) and psychological (*p* = 0.04) domains in this study sample.

**Conclusions:**

Improvements in OHRQOL may require a multidimensional approach with focus of social factors like education and behavioral factors like cigarette smoking. Also, improvement in OHRQOL might also lead to betterment of perceived overall health as they are interlinked.

## Background

The use of Quality of Life (QOL) measures for assessment of health effects, health outcomes, effectiveness of various health interventions are increasing. According to WHO, it is individuals’ perceptions of their position in life in the context of the culture and value systems in which they live, and in relation to their goals, expectations, standards and concerns [1]. Similarly, Oral Health-Related Quality of Life (OHRQOL) is defined by individual assessment of several oral health dimensions including physical dental function, tooth pain, psychological discomfort, and social impacts - all of which affect overall well-being [2]. The oral cavity has been described as “the window to general health” [3]. It is also an intersection of dentistry and medicine, semi-independent professions that share the same common goal of improving the health and quality of life of patients [4]. Oral health affects general health by causing considerable pain and suffering and by changing what people eat, their speech and their quality of life and well-being [5]. The relationship of oral health with various systemic conditions like atherosclerotic vascular disease, pulmonary disease, diabetes, and pregnancy-related complications that effect a large percentage of the population has been well studied [6]. Perceived dental health has shown to have a significant predictive effect on overall health perception and life satisfaction [7]. Thus, it seems plausible that Health Related Quality of Life (HRQOL) measures are associated with OHRQOL dimensions in Nepalese context as well.

Oral diseases including oral cancers, periodontal disease, dental caries, and tooth loss are linked to emerging chronic non-communicable diseases primarily because of common risk factors such as poor dietary habits, poor oral hygiene, and use of tobacco and alcohol [8]. Since, the Surgeon General’s seminal publication on smoking and health [9] in 1964, a plethora of additional information has emerged demonstrating a relationship between smoking and poor oral health [10]. The adverse effects of tobacco on oral health including both common and rare oral conditions and diseases have been reported in Nepal as well [11, 12]. Social determinants of oral health like literacy levels, access to dental care, age, economic status, etc., have also been established [13, 14]. However, reported information quantifying the effects of smoking and demographic characteristics on OHRQOL is inadequate.

With this background, this study was designed to find association of smoking and socio-demographic characteristics with OHRQOL and to determine association between OHRQOL and HRQOL in a sample of dental patients in Nepal.

## Methods

### Study design

A hospital based cross sectional study aimed to find association of smoking and socio demographic characteristics with OHRQOL and to determine association between OHRQOL and HRQOL among dental patients in Nepal.

### Study setting

The study was conducted among patients who visited Department of Oral and Maxillofacial Surgery of Nepal Medical College Teaching Hospital (NMCTH), Jorpati, Kathmandu during the data collection period i.e. December 2015–June 2016. This is a private teaching hospital with dental health insurance program only for staff members of the hospital.

### Selection criteria

The inclusion criteria for this study were new patients who visited Department of Oral and Maxillofacial Surgery of NMCTH for same dental treatment i.e. removal of tooth/teeth. Patients who did not have dental health insurance were a part of the study. Patients with severe pain and inability to respond to the study tools were excluded from the study.

### Operational definition for smokers

The study participants were divided into two groups i.e. *current smokers* who have smoked 100 cigarettes in his/ her lifetime and who currently smoke cigarettes. The other group comprised of *never smokers* who have never smoked, or who have smoked less than 100 cigarettes in his/her lifetime [15].

### Sample size and sampling procedure

The sample size was calculated using formula for two proportions where proportion having poor OHRQOL among nonsmokers (P1) and current smokers (P2) were taken as 0.14 and 0.24 respectively based on Brazilian study [16]. Initial calculation showed that 241 patients were required in each group. Finite population correction was applied as only 180 new cases visited the oral surgery department from December to June (6 months) of previous year. Thus, the corrected sample size was 105. After adding 15% to account for any errors in the study process, at least 125 study participants were required in each group for the study. Consecutive sampling [17] was then used to select study participants till the desired sample size of 250 i.e. 125 current smokers and 125 nonsmokers was achieved.

### Study tools

The study tool was a self-administered questionnaire comprising of information related to socio demographic variables, smoking status, HRQOL items assessed through World Health Organization Quality of Life Brief version (WHOQOL-Bref) and Oral health Impact Profile – 14 (OHIP-14) to assess OHRQOL.

WHOQOL-Bref has 24 items which constitute the four WHO QOL domains (physical, psychological, social, and environment) which has been translated to local language and validated in Nepalese settings for assessment of HRQOL [18]. The higher scores indicate better general HRQOL. The OHIP-14 index provides a comprehensive measure of self-reported dysfunction, discomfort and disability arising from oral conditions, the dimensions of which are based on Locker’s conceptual model of oral health. The responses are rated on a 5-point Likert scale: 0 = never; 1 = hardly ever; 2 = occasionally; 3 = fairly often; 4 = very often/every day. The OHIP-14 scores can range from 0 to 56 and are calculated by summing the ordinal values for the 14 items. The domain scores can range from 0 to 8. Higher OHIP-14 scores indicate worse and lower scores indicate better OHRQOL [19]. The OHIP-14 has been translated in local language and validated in Nepalese settings [20].

### Study variables

The independent variables of the current study are HRQOL scores, age, sex, educational status, current marital status, current status of earning and smoking status while the dependent variable is OHRQOL.

### Statistical analysis

Responses to each item of OHIP-14 were displayed through frequencies and percentages. Descriptive statistics like mean, median, standard deviation and inter quartile range were used to summarize total OHIP and subscale scores. Firstly, data distribution was checked for normality using the Kolmogorov - Smirnov tests. Both OHRQOL and HRQOL scores did not follow normal distribution. Thus, non-parametric tests were applied to test association between study variables. The OHIP scores (total and subscales) between never and current smokers were compared using Mann Whitney U test. The association of OHIP scores with socio demographic variables like sex, earning status, marital status, age and education categories was tested through Mann Whitney U test and Krushkal Wallis test with Dunn’s test. Due to non-normal distribution of the study data, further regression analyses and modelling were not performed. Correlation between OHIP-14 scores and domains of WHOQOL-Bref was tested with the help of Spearman Rank’s Correlation Coefficient. The level of significance was set at 5%.

## Results

The distribution of responses showed that about a quarter of the study patients reported pain in mouth (30.4%), self-consciousness (36.0%), tensed feeling (40.4%), unsatisfactory diet (29.6%) and embarrassment (28.8%) at a fairly often basis. Also, more than one third (37.6%) of patients very often felt uncomfortable while eating food (Table 1). The OHIP-14 mean total score was 21.71 out of the possible 56. The highest average value was recorded in the subscale of psychological discomfort followed by physical pain and physical disability while the lowest values were recorded for psychological disability and handicap (Table 2). It was found that in our study sample, current smokers had higher mean ranks compared to non- smokers in all the sub scales and total OHIP-14 scores. This difference was found to be statistically significant (*p* value < 0.05) in total OHIP scores and subscales of functional limitation, physical pain, social disability and psychological disability (Table 3)**.** The study results showed that similar mean OHIP scores were reported by both males and females. On the other hand, currently married and earning patients showed higher OHIP scores and thus, poorer OHRQOL. However, this difference of scores was not found to be statistically significant. The patients of the study reported higher mean OHIP scores as they grew older i.e. poorer OHRQOL in higher age groups especially among patients of ≥60 years of age. However, this trend was found to be insignificant. On the other hand, education was found to be significantly associated with OHRQOL. The result showed that compared to illiterate patients, the patients with ≥ high school education had significantly lower (*p* = 0.042) OHIP scores and thus, better OHRQOL (Table 4).

**Table 1:** Frequency distribution of responses for the items of OHIP-14 scores. (*n* = 250)

Items of OHIP-14	Never 0	Hardly ever 1	Occasionally 2	Fairly often 3	Very often 4
Difficulty in pronouncing word	115 (46%)	83 (33.2%)	18 (07.2%)	25 (10.0%)	09 (3.6%)
Taste has worsened	29 (11.6%)	152 (60.8%)	33 (13.2%)	20 (8.0%)	16 (6.4%)
Pain in mouth	22 (8.8%)	122 (48.8%)	21 (8.4%)	76 (30.4%)	09 (3.6%)
Uncomfortable eating food	31 (12.45)	79 (31.6%)	22 (8.8%)	24 (9.6%)	94 (37.6%)
Self – consciousness	28 (11.2%)	94 (37.6%)	30 (12.0%)	90 (36.0%)	08 (3.2%)
Tense feeling	36 (14.4%)	84 (33.6%)	21 (8.4%)	101 (40.4%)	08 (3.2%)
Unsatisfactory diet	13 (5.2%)	137 (54.8%)	22 (8.8%)	74 (29.6%)	04 (1.6%)
Interruption of meals	30 (12.0%)	139 (55.6%)	27 (10.8%)	49 (19.6%)	05 (2.0%)
Difficult to relax	16 (6.4%)	167 (66.8%)	20 (8.0%)	38 (15.2%)	09 (3.6%)
Feeling embarrassed	15 (6.0%)	131 (52.4%)	21 (8.4%)	72 (28.8%)	11 (4.4%)
Irritable with others	20 (8.0%)	170 (68.0%)	26 (10.4%)	23 (9.2%)	11 (4.4%)
Difficulty doing usual jobs	31 (12.4%)	154 (61.6%)	22 (8.8%)	11 (4.4%)	32 (12.8%)
Life less satisfying	26 (10.4%)	139 (55.6%)	22 (8.8%)	57 (22.8%)	06 (2.4%)
Totally unable to function	33 (13.2%)	172 (68.8%)	19 (7.6%)	15 (6.0%)	11 (4.4%)

**Table 2:** Descriptive statistics of total OHIP-14 scores and subscales. (*n* = 250)

OHIP variables	(Mean ± sd)	Median ± IQR
Psychological disability	1.43 ± 0.94	1 (1–2)
Social disability	2.78 ± 1.74	2 (2–4)
Handicap	1.51 ± 1.03	1 (1–3)
Physical disability	4.31 ± 2.18	3 (3–6)
Physical pain	4.0 ± 2.20	4 (2–6)
Functional limitation	2.29 ± 1.80	2 (1–3)
Psychological discomfort	5.40 ± 2.48	5 (3–7)
OHIP-14 total score	21.71 ± 8.94	20 (15–27)

**Table 3:** Comparison of subscales and overall OHIP-14 score among current and nonsmokers. (*n* = 250)

Variables	Current smokers (mean rank)	Nonsmokers (mean rank)	*P* value
Psychological disability	136.97	114.03	0.001*
Social disability	130.83	120.17	0.003*
Handicap	129.55	121.45	0.206
Physical disability	141.51	109.49	0.326
Physical pain	137.78	113.22	< 0.001*
Functional limitation	129.93	121.07	0.007*
Psychological discomfort	139.21	111.79	0.317
OHIP-14 total score	141.40	109.60	0.002*

*Statistically significant

**Table 4:** Association of total OHIP-14 with socio demographic variables. (*n* = 250)

Socio demographic variables	Categories (n)	OHIP-14 (mean ± sd)	Mean rank	*P* value
Age (in years)	15–30 (41)	19.63 ± 8.33	106.83	0.123
	30–45 (71)	21.01 ± 8.69	120.77	
	45–60 (81)	21.91 ± 9.76	124.23	
	≥60 (57)	23.7 ± 8.18	146.63	
Sex	Male (110)	21.82 ± 8.72	110	0.721
	Female (140)	21.63 ± 9.13	140	
Current marital status	Married (222)	21.97 ± 9.08	127.42	0.236
	Unmarried (28)	19.68 ± 7.50	110.27	
Current earning status	Earning (96)	22.11 ± 9.96	126.34	0.885
	Not earning (154)	21.46 ± 8.26	124.98	
Education	Illiterate (110)	22.89 ± 9.14*	110	0.042*
	Less than high school (50)	22.46 ± 9.42	50	
	≥ high school (90)	19.86 ± 8.17*	90	

*Statistically significant

The study showed that OHRQOL assessed through OHIP-14 and HRQOL assessed through WHOQOL-Bref are correlated. The OHIP scores are negatively correlated with all the four domains of WHOQOL-Bref with statistically significance in physical and psychological domains (*p* < 0.05). This indicates that poor OHRQOL was correlated with poor overall HRQOL in physical and psychological domains in our study sample (Table 5).

**Table 5:** Correlation between OHIP-14 and WHOQOL- Bref domain scores

OHIP - 14	WHOQOL-Bref domains	Correlation coefficient	*P* value
Total scores	Physical	- 0.154	0.015*
	Psychological	- 0.125	0.049*
	Social	- 0.078	0.221
	Environmental	- 0.122	0.055

*Statistically significant

## Discussion

### Distribution of OHRQOL among the dental patients

In the current study, the overall mean OHIP-14 score of the 250 dental patients was 21.71. The highest average value was recorded in the subscale of psychological discomfort followed by physical pain and physical disability while the lowest values were recorded for psychological disability and handicap. Similar findings were reported by an Iranian study where the mean score of OHIP-14 was 22.4 ± 8.2 among the older dental patients with highest score in the domain of psychological discomfort [21]. In a study done in India, the mean OHIP - 14 scores among people with Oral Sub Mucous Fibrosis was 19.10 and highest impact due to physical pain and psychological discomfort [22]. Another study done in Pakistan reported mean OHIP-14 scores of 13.59 among patients with hypodontia. Also, as the number of missing teeth increased, greater was the impact on psychological discomfort [23]. Another study reported that dental malocclusion has significant negative impact on OHRQOL, better OHIP-14 scores among patients who have undergone orthodontic treatment compared to the untreated control group [24]. This indicates that OHRQOL scores are dependent on type and severity of dental problems.

### Association of smoking with OHRQOL

In the present study, current smokers had poorer OHRQOL compared to nonsmokers. Similar to our study, a cross sectional study from India also reported higher OHIP-14 scores of smokers [25]. A national cohort of 87,134 Thai adults showed that smokers had worse OHRQOL in all dimensions of OHIP i.e. discomfort, speaking, swallowing, chewing, social interaction and pain [26]. Also, a Turkish study showed significant correlation of harmful habits including smoking with poorer OHRQOL compared to patients who had no harmful habits [27]. The negative impact of smoking on oral tissues leading to oral ill-effects can be held responsible for poorer OHRQOL among current smokers.

### Socio demographic determinants of OHRQOL

In the present study, major social determinant of OHRQOL was education i.e. illiterate patients reported poorer OHRQOL scores compared to patients with more than high school education. The other socio demographic characteristics like age, sex, marital status, earning status were not associated with OHRQOL in this study sample. Diverse findings have been reported in literature in this respect. A study from rural Nepal reported no relationship between sex and OHRQOL which was similar to our study. However, it also reported higher age to be associated with low OHRQOL which was not observed in our study [28]. Another study from India reported association of education with OHRQOL similar to our study [25]. A community-based study among elderly Iranians reported no significant difference in OHIP-14 scores according to gender, with significantly better OHRQOL scores among participants with academic education which corroborates with our study findings [21]. Studies from Brazil and Russia have also reported association of socio demographic characteristics like female gender, lower class, and rural household with poorer OHRQOL [29, 30]. These dissimilar findings suggest that socio-demographic determinants of OHRQOL are different in different settings.

### Correlation between OHRQOL and HRQOL

In the current study, OHRQOL was significantly correlated with HRQOL in physical and psychological domains. A study from Germany also reported significant correlation between OHRQOL and HRQOL in both physical and mental component scores [31]. Similar findings were reported by a study among patients with oral and oropharyngeal cancer patients which reported significant association of OHIP-14 scores in all domains of HRQOL assessed through SF-12 [32]. A Korean study also concluded that self – rating general health was positively associated with oral health [33]. Similarly, a study showed significant impact of oral health on health-related quality of life evaluated through modeling with “Health” as the central construct [34]. Another study on Belgian older adults also revealed that individuals who had poorer oral health had a higher risk of suffering from poor general health status. The prediction for general health predictors from oral health predictors in this study was around 80% which is pretty high [35].

Though the study tried to ensure comparability between the two groups and used validated study tools, there are various limitations as well. Firstly, since we used consecutive sampling technique from a single hospital, chances of selection bias and limited external validity cannot be ruled out. Secondly, inferences from this study need to interpreted carefully as they are based on non-parametric tests.

## Conclusions

This study showed relationship of smoking and education with OHRQOL and correlation between OHRQOL and HRQOL in a Nepalese sample. These study findings are important for both oral health professionals and public health experts. Improvements in OHRQOL require multidimensional approach including addressing factors like education and cigarette smoking. Also, improvement in OHRQOL might also lead to betterment of perceived overall health as they are interlinked.

## Data Availability

The datasets used and/or analyzed during the current study are available from the corresponding author on reasonable request.

## Abbreviations

QOL: Quality of Life
HRQOL: Health Related Quality of Life
OHRQOL: Oral Health Related Quality of Life
NMCTH: Nepal Medical College Teaching Hospital
WHOQOL-Bref: WHO Quality of Life BREF
OHIP: Oral Health Impacts Profile

## References

[1] WHOQOL Group (1996). What quality of life? World Health Organization quality of life assessment. World Health Forum.

[2] Brennan DS, Spencer AJ (2004). Dimensions of oral health related quality of life measured by EQ-5D+ and OHIP-14. Health Qual Life Outcomes.

[3] Seymour GJ (2007). Good oral health is essential for good general health: the oral-systemic connection. Clin Microbiol Infect.

[4] Alpert PT (2017). Oral health: the oral-systemic health connection. Home Health Care Manag Pract.

[5] Sheiham A (2005). Oral health, general health and quality of life. Bull World Health Organ.

[6] Kuo LC, Polson AM, Kang T (2008). Associations between periodontal diseases and systemic diseases: a review of the inter-relationships and interactions with diabetes, respiratory diseases, cardiovascular diseases and osteoporosis. Public Health.

[7] Locker D, Clarke M, Payne B (2000). Self-perceived oral health status, psychological well-being, and life satisfaction in an older adult population. J Dent Res.

[8] Petersen PE, Bourgeois D, Ogawa H, Estupinan-Day S, Ndiaye C (2005). The global burden of oral diseases and risks to oral health. Bull World Health Organ.

[9] Department of Health and Human Services (US) (1964). Reducing the health consequences of smoking: a report of the Surgeon General.

[10] Office of the Surgeon General (US) (2004). The health consequences of smoking: a report of the Surgeon General.

[11] Reibel J (2003). Tobacco and oral diseases. Update on the evidence with recommendations. Med Princ Pract.

[12] Dhami B, Shrestha P, Humagain M (2013). Effect of cigarette smoking on periodontal health status in Nepalese population: a cross-sectional study. J Nepal Dent Asssoc.

[13] Tellez M, Zini A, Estupiñan-Day S (2014). Social determinants and oral health: an update. Curr Oral Health Rep.

[14] Petersen PE, Kwan S (2011). Equity, social determinants and public health programmes – the case of oral health. Community Dent Oral Epidemiol.

[15] Centers for Disease Control and Prevention, National Center for Health Statistics: Glossary of National Health interview survey, 2017. [Available from : https://www.cdc.gov/nchs/nhis/tobacco/tobacco_glossary.html].

[16] Gabardo MCL, Moysés SJ, Moysés ST, Olandoski M, Olinto MTA, Pattussi MP (2015). Social, economic, and behavioral variables associatedwith oral health-related quality of life among Brazilian adults. Ciênc Saúde Coletiva.

[17] Mathieson K. Making sense of biostatistics: types of nonprobability sampling. J Clin Res Best Pract. 2014;10(10).

[18] Giri S, Neupane M, Pant S, Timalsina U, Koirala S, Timalsina S (2013). Quality of life among people living with acquired immune deficiency syndrome receiving anti-retroviral therapy: a study from Nepal. HIV AIDS.

[19] Slade GD, Spencer AJ (1994). Development and evaluation of the oral health impact profile. Community Dent Health.

[20] Vikram M, Singh VP. Translation and validation of the Nepalese version of oral health impact profile (OHIP-14) questionnaire. Oral Biol Dent, Herbert Open access Journals. 2014.

[21] Mottallebnejad M, Mehdizadeg S, Najafi N, Sayyadi F (2015). The evaluation of oral health-related factors on the quality of life of the elderly in Babol. Contemp Clin Dent.

[22] Jena AK, Rautray S, Mohapatra M, Singh S (2018). Oral health – related quality of life among male subjects with oral submucous fibrosis in a tertiary care hospital. Indian J Public Health.

[23] Chhetri S, Khan MSU, Yazdanie N (2017). Comparison of oral health related quality of life (OHRQOL) in hypodontia patients and patients with acquired missing teeth. J Nobel Med Coll.

[24] Demirovic K, Habibovic J, Dzemidzic V, Tiro A, Nakas E (2019). Comparison of oral health-related quality of life in treated and non-treated orthodontic patients. Med Arch.

[25] Batra M, Shah AF, Dany SS, Rajput P (2017). Determinants related to oral health-related quality of life among subjects attending a dental institute in Morabad city – a cross sectional study. J Indian Assoc Public Health Dent.

[26] Yiengprugsawan V, Somkotra T, Seubsman S, Sleigh AC (2011). Oral health-related quality of life among a large national cohort of 87,134 Thai adults. Health Qual Life Outcomes.

[27] Caglayan F, Altun O, Miloglu O, Kaya MD, Yilmaz AB (2009). Correlation between oral health-related quality of life (OHQOL) and oral disorders in a Turkish patient population. Med Oral Patol Oral Cir Bucal.

[28] Agrawal SK, Dahal S, Shrestha A, Bhagat TK (2017). Assessment of oral health impact profile (OHIP −14) among villagers of Jyamirgadi VDC, Nepal: a cross-sectional study. Eur J Biomed Pharm Sci.

[29] Ulinksi KGB, Nacimento MA, Lima AMC (2013). Factors related to oral health –related quality of life of independent Brazilian elderly. Int J Dentistry.

[30] Drachev N, Brenn T, Trovik TA (2018). Oral health-related quality of life in young adults: a survey of Russian undergraduate students. Int J Environ Res Public Health.

[31] Zimmer A, Bergmann N, Gabrun E, Barthel C, Raab W, Ruffer JU (2010). Association between oral health-related and general health-related quality of life in subjects attending dental offices in Germany. J Public Health Dent.

[32] Barrios R, Tsakos G, Gil-Montoya JA, Montero J, Bravo M (2015). Association between general and oral health-related quality of life in patients treated for oral cancer. Med Oral Patol Oral Cir Bucal.

[33] Sim SJ (2014). Association between oral health status and perceived general health (Euroqol – 5D). J Dent Hyg Sci.

[34] Zucoloto ML, Maroco J, Compos JADB (2016). Impact of oral health on health-related quality of life: a cross-sectional study. BMC Oral Health.

[35] Tran TD, Hofmann SK, Duyck J (2018). Association between oral health and general health indicators in older adults. Nature/Sci Rep.

